# Single-Charge Tunneling in Codoped Silicon Nanodevices

**DOI:** 10.3390/nano13131911

**Published:** 2023-06-22

**Authors:** Daniel Moraru, Tsutomu Kaneko, Yuta Tamura, Taruna Teja Jupalli, Rohitkumar Shailendra Singh, Chitra Pandy, Luminita Popa, Felicia Iacomi

**Affiliations:** 1 Research Institute of Electronics, Shizuoka University, 3-5-1 Johoku, Naka-ku, Hamamatsu 432-8011, Japan; 2 Faculty of Physics, Alexandru Ioan Cuza University of Iasi, 11 Carol I Blvd., 700506 Iasi, Romania

**Keywords:** doping, codoping, silicon, phosphorus, boron, single-electron tunneling, nanostructures, dopant-induced quantum dots

## Abstract

Silicon (Si) nano-electronics is advancing towards the end of the Moore’s Law, as gate lengths of just a few nanometers have been already reported in state-of-the-art transistors. In the nanostructures that act as channels in transistors or depletion layers in *pn* diodes, the role of dopants becomes critical, since the transport properties depend on a small number of dopants and/or on their random distribution. Here, we present the possibility of single-charge tunneling in codoped Si nanodevices formed in silicon-on-insulator films, in which both phosphorus (P) donors and boron (B) acceptors are introduced intentionally. For highly doped *pn* diodes, we report band-to-band tunneling (BTBT) via energy states in the depletion layer. These energy states can be ascribed to quantum dots (QDs) formed by the random distribution of donors and acceptors in such a depletion layer. For nanoscale silicon-on-insulator field-effect transistors (SOI-FETs) doped heavily with P-donors and also counter-doped with B-acceptors, we report current peaks and Coulomb diamonds. These features are ascribed to single-electron tunneling (SET) via QDs in the codoped nanoscale channels. These reports provide new insights for utilizing *codoped silicon nanostructures* for fundamental applications, in which the interplay between donors and acceptors can enhance the functionalities of the devices.

## 1. Introduction

Silicon (Si) has been widely used in electronics due to its abundance, low cost for processing, and versatile properties. However, the electrical properties of intrinsic silicon limit its use and doping has been generally adopted to change the conductivity by the intentional introduction of substitutional impurities into the silicon lattice [1]. This approach allowed the formation of *pn* junctions as part of the design of a variety of electronic devices, such as *npn* or *pnp* transistors, as well as tunnel diodes or *pn*/*pin* diodes. As Moore’s law [2] approaches the end of line, with the critical dimensions of transistors becoming 10 nm or less, the requirements imposed on the *pn* junctions, in terms of doping concentration and junction abruptness, become limiting factors in the design and fabrication of nanoscale devices [3,4].

Recently, junctionless transistors [5] have been proposed and demonstrated to overcome the technological challenges imposed by the necessity to control the doping profile in nanoscale. Such junctionless transistors have a relatively simple design, with uniform doping at high concentration across the channel. The high conductance due to the heavy *n*-type (or *p*-type) doping can be controlled because the channel is a nanowire, and the surrounding gate can deplete such a Si channel [6]. In state-of-the-art junctionless transistors, full depletion corresponds to the off-state [7]. In nanoscale transistors doped with lower concentrations, it has been reported that even individual dopants can control the low-temperature transport as quantum dots (QDs), either for donors [8,9,10,11,12,13] or for acceptors [14,15]. Under such conditions, doping with one type of dopant has been shown to have an impact on optoelectronics from diverse aspects [16], including for single-photon detection [17].

The directions outlined above illustrate the importance of both types of doping (*n*-type or *p*-type), as well as the relevance of the high doping concentration. So far, however, codoping has been rarely considered as a significant technology for applications in electronics, and then mainly because donors and acceptors facing each other in *pn* junctions are expected to induce resonant tunneling current [18]. Mostly, codoping found applications in the fields of photonics and optoelectronics [19,20]. Considering the interactions between donors and acceptors, it can be implied that codoping can be a valuable process to consider for tuning the electrical properties of nanoscale Si. There are different elements that can be used as dopants in Si, such as phosphorus (P), arsenic (As), antimony (Sb), and bismuth (Bi) as donors and, respectively, boron (B), aluminum (Al), gallium (Ga), and indium (In) as acceptors [21]. It is generally accepted that P and B are technologically suitable as dopants in Si due to their relatively shallow ground-state energy states and controllability of diffusion. In fact, it is well known that, by controlling the concentration and distribution of impurities (P donors and B acceptors), the electrical conductivity and refractive index of Si can be tailored for specific applications [22]. Codoping Si with P and B dopants has also been shown to enhance the carrier mobility, dramatically modify the recombination rate, and improve the light absorption and emission properties of Si-based devices [23].

Beyond these effects of codoping on the macroscopic properties of Si, its effect on the microscopic potential landscape in nanoscale devices can be even more dramatic. Due to the inherent randomness of dopant distribution, clusters of donors and/or acceptors can induce local potential wells. These potential wells work as quantum dots (QDs) capturing charges and mediating transport of carriers one by one. A critical effect in such devices is the compensation between donors and acceptors, which may allow the formation of clusters (i.e., QDs) even though doping concentrations of both types of dopants are high [24].

Here, we provide a perspective on the importance of codoping in promoting single-charge tunneling transport in nanoscale Si devices. This article considers two fundamental devices, diodes and transistors, focusing on devices fabricated in thin silicon-on-insulator (SOI) layers, presenting few results that can be related to codoping. The aim is to open new pathways for implementing codoping as a key technology in future nanoscale devices, enhancing single-charge tunneling features specifically induced by codoping itself.

**Figure 1:**
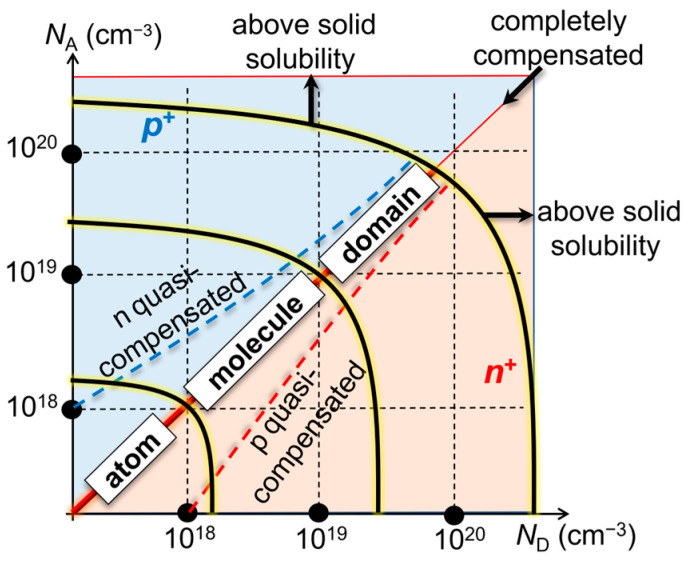
An overview of the codoped-Si space defined by the two doping concentrations, *N*_D_ and *N*_A_, in a wide range. As *N*_D_ and *N*_A_ change together, one can explore different natures of interactions between donors and acceptors: (i) “atom” nature (for inter-dopant distance *d* >> 2*r*_B_, with *r*_B_ being the Bohr radius for a dopant in Si); (ii) “molecule” nature (for *r*_B_ < *d* < 2*r*_B_); (iii) “domain” nature (for *d* < *r*_B_). Solid solubility imposes the limits on how far doping concentrations can be extended in this space.

Figure 1 shows an overview of the space defined by the two doping concentrations, *N*_D_ and *N*_A_, ranging from 10^17^ cm^−3^ (corresponding to inter-dopant distances on the order of 20 nm) to more than 10^20^ cm^−3^ (close to the solid solubility limits, corresponding to inter-dopant distances on the order of 2 nm). Assuming that this space is defined in a thin nanoscale Si layer, one can delineate several regimes that reveal different natures of dopants: (i) “atom” nature, where each individual dopant-atom is practically isolated from the others—at low concentrations, well below the metal-insulator transition (MIT) [25,26]; (ii) “molecule” nature, where several dopants can be found coupling to each other into “clusters” (with molecule-like behavior)—at medium concentrations, slightly below or above MIT [27,28]; (iii) “domain” nature, where a larger number of dopants dominantly define *n*-type or *p*-type zones (domains) [29] due to the uncorrelated distribution of opposite-polarity dopants—at higher concentrations, approaching the solid-solubility limits. The mid-line in this space corresponds to the full (complete) compensation. As one deviates from the mid-line, a quasi-compensated (or partially compensated) regime can be expected [30,31,32]. When the concentrations are strongly imbalanced, Si can be simply treated as *n* or *p*, with only minor disturbance from the opposite-polarity dopants.

A few results will be provided for different devices with codoped active areas, simply to illustrate the emergence of such natures in single-charge tunneling transport.

## 2. Fabrication of Codoped Nanoscale-Si Devices

In order to explore the effects of codoping on nanoscale devices, SOI substrates provide the most suitable platform because the top Si layer can be scaled down in all three dimensions. Thinning down, either intentionally by sacrificial oxidation and etching, or as a natural result of device processing, can allow the formation of films in the range of 10 ± 5 nm with relative ease. It should be noted, however, that flatness must be preserved if the effect of dopants must be clearly distinguished from the accidental effect of roughness in the formation of QDs [33,34]. Lateral dimensions (width and length) of the active regions can be defined by techniques such as electron beam lithography (EBL), with precision on the order of several tens of nm or less. Similarly to the point made above, line edge roughness (LER) must be controlled to avoid fluctuations that can result in the accidental formation of an undulated potential landscape [35].

**Figure 2:**
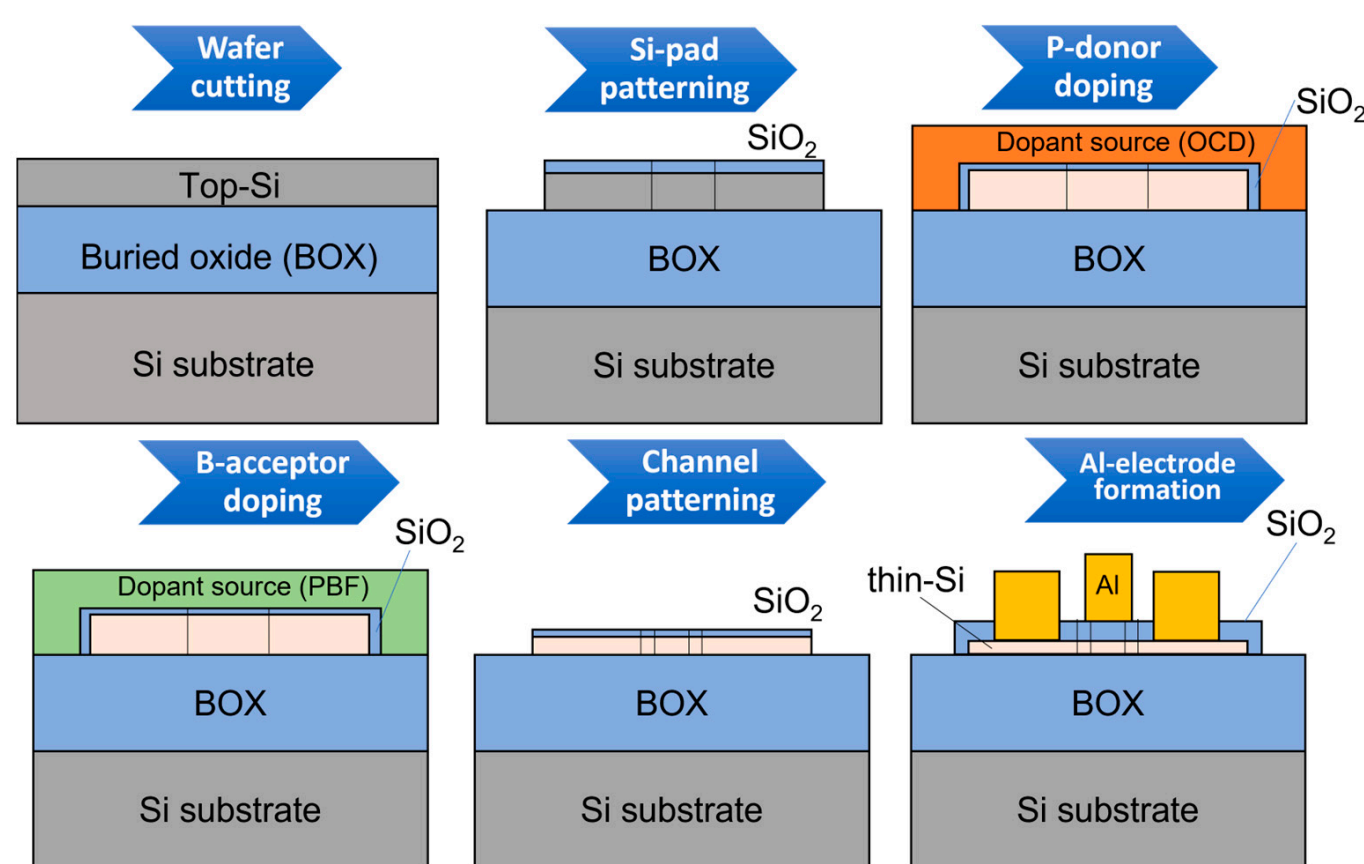
A typical fabrication process flow for SOI codoped nanodevices (doping masks are needed for *pn* diodes and a top Al gate is formed for transistors). The steps are: wafer cutting (and an initial thorough cleaning process), Si-pad patterning, P-doping, B-doping (both described in the text), nano-channel patterning, and, finally, Al-electrode formation (by a lift-off process).

In this work, we will overview results obtained from different types of codoped devices, but the SOI platform and the basic fabrication steps are common to all. Therefore, a typical fabrication flow will be described in the following. Figure 2 illustrates schematically the main fabrication steps for SOI devices, either SOI lateral *pn* (and *pin*) diodes or codoped SOI field-effect transistors (SOI-FETs).

As shown in Figure 2, fabrication starts from SOI layers with thicknesses in the range of 50–70 nm and with a buried oxide (BOX) with thickness in the range of 150–200 nm. The substrate is weakly-doped *p*-type Si(100) with a concentration *N*_A_ ≈ 1.5 × 10^15^ cm^−3^. In most cases, the BOX film is sufficiently thick to ensure electrical isolation of the top Si(100) layer from the substrate, so that the transport through the top layer is independent of substrate Si (unless specifically used as a back gate). The SOI layer is thinned down to the target starting thickness by sacrificial oxidation (typically, dry oxidation at 900–950 °C for 10–20 min, resulting in oxide layers of thicknesses on the order of 10 nm), followed by etching in a diluted-HF solution. This process is expected to induce minimum roughness since only a few nm of the Si layer are consumed during every sacrificial-oxidation step. Large Si pads are then defined for making the contacts for metal electrodes. In our recent fabrication approach, it is at this stage that doping processes are introduced because of the ease of observation and the final step is reserved for the fine patterning of the active area of the devices, after doping.

There are two types of doping that are successively done: *n*-type (with P-donors from spin-coated solution, OCD59230, containing P_2_O_5_) and *p*-type (with B-acceptors from another spin-coated solution, PBF6M-10, containing B_2_O_3_). The order in which these doping processes are done can be arbitrary, but it also depends on the amount of thermal budget that dopants will be subjected to. Typically, higher temperatures are required for doping P-donors than for doping B-acceptors at comparable concentrations, which means that carrying out the B-doping first will expose the B-acceptors to a significant thermal budget during the P-doping process. Although this is less important when codoped transistors (basically, junctionless) are formed, this extended diffusion length is detrimental for the formation of abrupt *pn* junctions. Therefore, a usual sequence starts with P-doping, followed by B-doping. Before both doping processes, an ultrathin layer of SiO_2_ (~1.0 ± 0.5 nm) is thermally grown by dry oxidation at 650 °C for 10 min in order to further protect the SOI surface from direct exposure to the doping source, which may induce unnecessary roughness. In addition, masks are used to protect the regions of SOI that are not supposed to be doped, as follows: (i) a SiO_2_ mask of 10–30 nm against P-doping; (ii) a Si_3_N_4_ mask of 10–20 nm against B-doping. These masks allow the formation of *pn* junctions, consistent with conventional processes in very large-scale integration (VLSI) technology [36].

For both types of doping, a two-step process is used. First, after spin-coating the doping solution onto the SOI surface, protected by the ultrathin SiO_2_ layer, pre-deposition is carried out at 600 °C for 30 min in N_2_ atmosphere for P-doping and, respectively, in O_2_ atmosphere for B-doping. Pre-deposition for B-doping is done in O_2_ atmosphere in the present samples in order to allow the removal (burning) of the solvent from the spin-coated PBF film, which contains a polymer. Second, drive-in is carried out at temperatures ranging from 800 °C to 1050 °C for various times. Both drive-in time and temperature affect significantly the doping concentration, so they are chosen based on systematic testing. After the completion of the doping process, the surface is cleaned by sequences of H_2_SO_4_:H_2_O_2_ (4:1) and diluted HF, HF:DIW, deionized water (1:20) solutions. Measurements of thickness are taken with a thickness monitor (FE-3000, Otsuka Electronics) and resistivity is measured at room temperature by a four-point probe technique (K-705RS, Kyowa Riken). These measurements (on reference samples) allow the estimation of the doping concentrations to be within about ±20% of the average value.

It is important to note, at this stage, that the drive-in time must be short enough to ensure abrupt junctions (in the case when *pn* diodes are fabricated), but also long enough to ensure that dopants diffuse all the way to the bottom of the SOI layer (usually a few tens of nm). A suitable compromise must be achieved by testing, with secondary ion mass spectrometry (SIMS) as a powerful evaluation technique for addressing this issue.

Once codoping is completed, the fabrication flow proceeds to nano-patterning. For that, a thin SiO_2_ film is grown, and thin resist is spin-coated onto the surface. An EBL exposure with low current and high resolution is used to define the patterns, and reactive ion etching (RIE) is used to etch away the unwanted Si film after careful development of the resist. Since RIE etching preserves the abruptness of the side walls, without introducing large LER (and without directly affecting the top SOI surface), it can be assumed that this step can be sufficiently controlled to maintain roughness within reasonable limits.

The final stages of the fabrication simply aim to passivate the surfaces appropriately, to form the gate oxide, if necessary, and to define the metal electrodes. Gate oxide is also formed by dry oxidation (at least the first few nm), in order to preserve the quality of the Si/SiO_2_ interface. This step is usually followed by annealing in H_2_:N_2_ forming gas for passivating some amount of the dangling bonds present at the interface. Electrodes are defined by a lift-off process, in two steps, first to open the contact holes to make ohmic contacts, and second to deposit Al (typically, with a thickness in the range of 200–300 nm) by vacuum evaporation. The lift-off process is completed by acetone spray.

**Figure 3:**
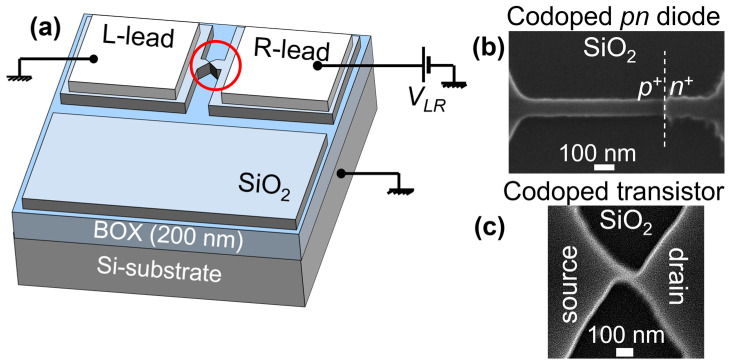
(**a**) A typical SOI device layout (top gate should cover the channel in the case of a transistor), where the top-Si layer contains the nanostructures that are the active regions between the left and right leads (*p*^+^-lead and *n*^+^-lead in the case of a *p*^+^*n*^+^ diode, or source and drain in the case of a transistor). (**b**) SEM image of a *p*^+^*n*^+^ diode with the junction formed towards the right side of the nanowire. (**c**) SEM image of the nanoscale channel of a codoped SOI-FET.

A simplified device structure is shown in Figure 3a. Scanning electron microscope (SEM) images of the central region of each device (transistor and diode) are shown in Figure 3b,c. Figure 3b shows a *p*^+^*n*^+^ diode formed in a 1-µm-long SOI nanowire (with both *N*_D_ and *N*_A_ on the order of 10^20^ cm^−3^, but *N*_D_ > *N*_A_). Figure 3c shows a codoped SOI-FET (before gate formation) with a short and narrow channel (*W*, *L* on the order of ~50 nm). It can be observed from these images that edges and surfaces look smooth enough to assume that roughness does not dramatically affect the electrical characteristics.

Electrical characterization is carried out in a vacuum chamber of a probing system, using semiconductor precision parameter analyzer (typically Keysight, B1500A). Measurements are mostly carried out at low temperatures (*T* ≈ 8.5 ± 0.5 K), with a noise level on the order of 10~100 fA. In this work, only low-temperature results will be displayed.

## 3. Results

Low-temperature current-voltage (*I*-*V*) measurements of codoped nanodevices, fabricated as described above, reveal the transport behavior under the conditions of a narrow Fermi-Dirac distribution of carriers, which helps minimize the effects of thermally-activated carriers. Such carriers may hinder the observation of the quantum-tunneling features. On the downside, low-temperature increases the resistivity of the material, in particular due to the freeze-out effect, which is significant at temperatures below 100 K [37]. The temperature of the measurements shown next is around 8 K, which corresponds to the thermal energy, *k*_B_*T* ≈ 0.7 meV, where *k*_B_ is the Boltzmann constant.

The results presented here are just examples of characteristics that can be ascribed to the effects of codoping, although further analysis is required to fully clarify this assignment. Due to the early stage of this research, this work aims to highlight the fact that more experimental and theoretical analysis is certainly demanded. The results are presented first for nanoscale *pn* diodes, in which the depletion layers may contain QDs and discrete energy states that can work as stepping-stones for band-to-band tunneling (BTBT) transport [38,39,40]. Then, results are presented for codoped SOI-FETs, which function similarly to junctionless transistors [5,6,7], with the key difference that codoping may be more effective than single-type doping [41] for the formation of QDs, thus likely promoting single-electron tunneling (SET).

### 3.1. Transport in (Codoped) SOI Nanoscale Diodes

In order to ensure that *pn* diodes are actually formed in nanostructures in the lateral layout proposed here, an overlap region is specifically designed in which both P-doping and B-doping are carried out. This overlap region is designed to be 500 nm or less within the nanowire (usually, with a length of 1000 nm), but possible mismatch (shift) of the doping masks may affect this overlap. Nevertheless, as shown in Figure 3b, it is confirmed that the overlap region is formed, with the *pn* junction appearing near the edge of the P-doping mask. This is because, in our devices so far, *N*_D_ > *N*_A_ (for the device described next, *N*_D_ ≈ 2.5 × 10^20^ cm^−3^ and *N*_A_ ≈ 1.5 × 10^20^ cm^−3^ after the typical two-step doping with the drive-in conditions: 975 °C, 10 min for P-doping and, respectively, 925 °C, 3 min for B-doping). The codoped overlap not only guarantees the formation of the *pn* junction, but it also additionally reduces the dimensions of the nanowire (on the right side of the junction).

For most of the devices in this batch, the *I*-*V* characteristics are significantly different from regular *pn* diodes, in which one expects that the forward-bias regime is dominated by the thermally-activated current, while the reverse-bias regime has practically no current until junction breakdown is observed [42]. In the case of the present devices, designed as *p*^+^*n*^+^ diodes, the forward-bias regime contains not only the thermally-activated current, but also large current steps and humps at lower voltages. At the lowest voltages (*V*_p_ ≤ 100 mV), current is typically low (comparable to noise level), but in a fraction of devices negative differential conductance (NDC) features can be observed. In the reverse-bias regime, current is generally larger than in the forward-bias regime (at least for small biases).

**Figure 4:**
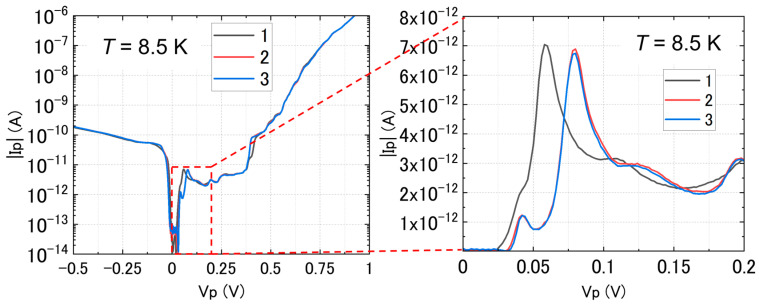
*I*-*V* characteristics at low temperature (*T* ≈ 8.5 K) for a *p*^+^*n*^+^ SOI diode, shown by the |*I*_p_| (absolute value of current in the device) as a function of the bias, *V*_p_. An NDC peak and a region of excess current, containing several current steps, are observed before the onset of the thermally-activated current component in the forward-bias regime. In the reverse-bias regime, current sharply increases at small *V*_p_, followed by a slower increase at larger *V*_p_. Zoom-in inset (right): linear-scale plot of the forward-bias regime at small *V*_p_ for three consecutive measurements, showing the stabilization of the *I*-*V* characteristics.

Figure 4 shows a typical result at low temperature (*T* ≈ 8.5 K) from the set of devices that exhibit NDC in the forward-bias regime, with the consecutive measurements displayed for reference. The zoom-in on the right provides a more detailed view of the behavior at small forward biases. It can be seen that the first curve (labeled “1”) is slightly different from the subsequent curves (labeled “2” and “3”), but the characteristics are stabilized afterwards. A large NDC peak (with a peak-to-valley ratio, PTVR, of about 3) is observed, along with smaller current peaks superimposed on the larger envelope. Such features have been already reported in our previous work [39], suggesting the possibility of BTBT transport mediated by donor-induced and acceptor-induced energy states present in the depletion layer. However, a small gap of ≈30 mV can also be noticed for these conditions. In the reverse-bias regime, the current sharply increases until *V*_p_ becomes about −100 mV, then follows a steady exponential growth.

**Figure 5:**
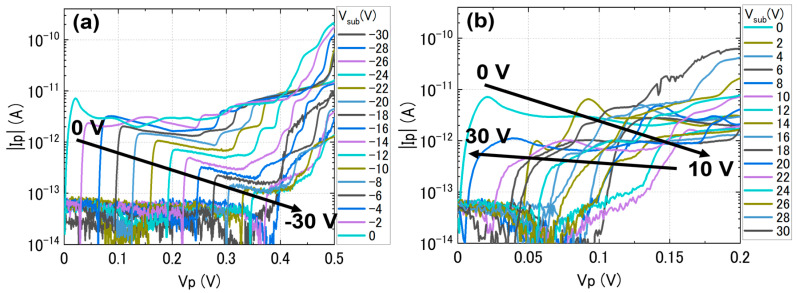
*I*_p_-*V*_p_ characteristics (*T ≈* 8.5 K) for the same *p*^+^*n*^+^ SOI diode, shown as a function of *V*_sub_ for: (**a**) negative *V*_sub_ range (0 ~ −30 V, in 2 V steps); (**b**) positive *V*_sub_ range (0 ~ +30 V, in 2 V steps). Arrows broadly indicate the directions of the shifts of the NDC peak, which is systematic in (**a**), but back-and-forth in (**b**).

The small gap observed at low biases becomes a point of interest, since it can be correlated with the Coulomb gap reported for SET devices. Since our *p*^+^*n*^+^ diodes do not have a top gate, we use the Si-substrate as a back gate to change the potential in the top-Si layer. It is expected that the larger Si-pads and the extensions leading to the depletion layer are less affected by the substrate voltage (*V*_sub_) because of larger sizes and/or higher effective doping concentrations. Therefore, *V*_sub_ may affect mostly the depletion layer (where *N*_D_ ≈ *N*_A_, with a higher effect of compensation between donors and acceptors). When *V*_sub_ changes in a wide range (from −30 V to +30 V), the *I*-*V* characteristics systematically change at first, followed by a more irregular behavior at large positive *V*_sub_. For clarity, the dependence on *V*_sub_ is displayed in Figure 5a,b for the negative *V*_sub_ range and, respectively, for the positive *V*_sub_ range, in both cases with a 2 V successive change of *V*_sub_. From Figure 5a, it can be observed that the NDC peak gradually shifts rightwards by about 30 mV (in *V*_p_) for every 2 V change in *V*_sub_, with these shifts becoming smaller at larger *V*_sub_. This change is also accompanied by a significant reduction of the NDC current level by more than one order of magnitude. In addition, the step structure observed at higher *V*_p_ also changes noticeably. The situation is more complex for the positive *V*_sub_ range, where the NDC current changes without an obvious systematic trend and the current level fluctuates, ending up when *V*_sub_ = 30 V (maximum tested) being comparable to the case when *V*_sub_ = 0 V (starting point), with less than one order of magnitude decrease in current.

**Figure 6:**
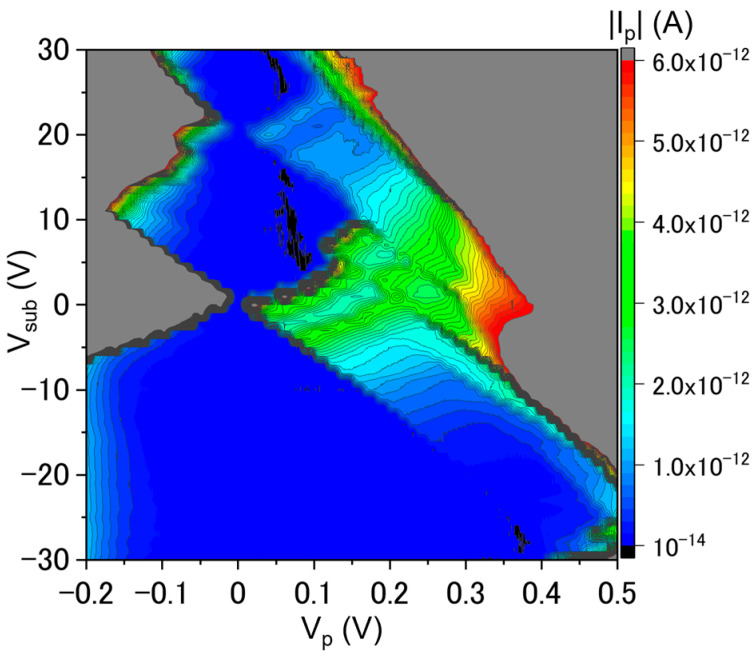
Stability diagram (plot of |*I*_p_| in the *V*_p_-*V*_sub_ space) at *T ≈* 8.5 K for the same *p*^+^*n*^+^ diode as shown in Figure 4 and Figure 5. Diamond-shaped regions are observed more prominently for positive *V*_sub_ (blue color corresponds to |*I*_p_| < 1 pA).

These results clearly illustrate the fact that *V*_sub_ can modify the potential in the top-Si layer, although the behaviors of the device in opposite polarities of *V*_sub_ appear to be different. The emergence of a larger gap (even fluctuating) at small biases as *V*_sub_ changes (as a back gate) is suggestive of the occurrence of Coulomb blockade in such a device. For confirming this behavior, changes are monitored by a stability diagram (plot of |*I*_p_| in the space defined by *V*_p_ and *V*_sub_) measured at *T* ≈ 8.5 K, as shown in Figure 6.

In the stability diagram, the low-current regions (blue regions around *V*_p_ = 0 V) correspond to currents |*I*_p_| < 1 pA. The systematic trend seen in Figure 5a for negative *V*_sub_ can now be visualized as a shift of the edge of the low-current region as *V*_sub_ changes (with some deviation at higher negative values of *V*_sub_). On the other hand, for positive *V*_sub_, the irregular trend seen in Figure 5b can now be visualized as clear diamond-shaped low-current regions in Figure 6. Although there is a complex structure embedded in these regions, the features are similar to the Coulomb diamonds typically reported for SET transistors [43,44]. These features are intriguing in terms of the possibility of observing single-charge BTBT transport. A more detailed interpretation will be provided in the Discussion section.

### 3.2. Transport in Codoped SOI Nanoscale Transistors

Codoped SOI nano-transistors have a simpler fabrication process in terms of doping, since no masks are used for selective doping of the devices. Instead, a uniform (non-selective) doping approach is taken, with P-doping followed by B-doping in the entire Si layer. For the devices shown here, the doping conditions for the drive-in process are somewhat changed as compared to the conditions for the *p*^+^*n*^+^ diodes, choosing to increase significantly the drive-in time, but reduce generally the temperature to further preserve surface flatness: 860 °C, 20 min for P-doping and, respectively, 960 °C, 5 min for B-doping, resulting in the concentrations: *N*_D_ ≈ 2.0 × 10^20^ cm^−3^ and *N*_A_ ≈ 0.5 × 10^20^ cm^−3^. These differences aimed to ensure also that doping is done in the entire depth of the top-Si layer (not only near the surface). The transistors are completed with an Al gate (along with source and drain) electrodes, with a thickness of about 250 nm, formed by a lift-off process. The gate oxide has been thermally grown as *t*_ox_ ≈ 10 ± 1 nm. Care was taken to ensure the formation of ohmic contacts for the source and drain electrodes.

**Figure 7:**
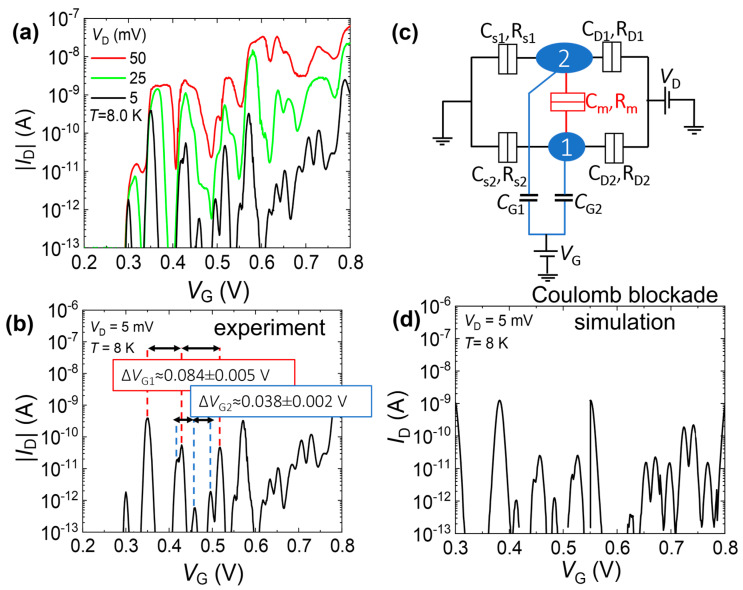
An example of electrical characteristics for a codoped nanoscale SOI-FET with channel designed as a point contact (design parameters: *L* = 0 nm; *W* = 50 nm), in comparison with simulations. (**a**) *I*_D_-*V*_G_ characteristics (at *T ≈* 8.0 K) as a function of source-drain bias (*V*_D_). (**b**) Analysis of the inter-peak periods (Δ*V*_G1_, Δ*V*_G2_) from the experimental low-bias data, assuming that the peaks are due to two different QDs. (**c**) Equivalent circuit illustrating two parallel QDs (with some mutual tunnel-coupling between them, described by C_m_, R_m_). (**d**) *I*_D_-*V*_G_ characteristics simulated based on the orthodox theory of Coulomb blockade for the circuit in (**c**) (tuned parameters are: C_G1_ = 2.3 aF, C_G2_ = 4.5 aF, C_S_ = C_D_ = C_m_ = 1.0 aF).

Figure 7a shows the electrical characteristics at low temperature (*T* ≈ 8.0 K) for one such codoped SOI-FET, designed nominally as a point contact (i.e., having the channel with designed dimensions *L* = 0 nm; *W* = 50 nm). The channel becomes elongated after patterning and further processing, with final dimensions: *L* ≈ 100 nm; *W* ≈ 30 nm, as seen in Figure 3c. The dependence on *V*_D_ is also shown in Figure 7a from 5 mV to 50 mV. For *V*_D_ = 5 mV (transport window slightly larger than the thermal energy at the measurement temperature), isolated current peaks can be observed. The peaks are not perfectly periodic, but rather contain two different periodicities. This is illustrated in Figure 7b, where (ignoring the first current peak) one can identify two sets of peaks with two distinct periods (Δ*V*_G1_ ≈ 0.084 V and Δ*V*_G2_ ≈ 0.038 V). The irregular current levels for different peaks also suggest that there are likely two parallel (weakly coupled) paths formed by two different QDs. The equivalent circuit for this model is shown in Figure 7c, with one of the QDs larger than the other, consistent with the simple model that *C*_Gi_ = *e*/Δ*V*_Gi_, with *e* the elementary charge and *i* (1,2) the index of the QD. The simulated *I*_D_-*V*_G_ characteristics for similar conditions as in the experiment, carried out under the assumption that transport occurs within the orthodox theory of Coulomb blockade [44], are shown in Figure 7d. By tuning the parameters of this equivalent circuit, in particular *C*_G1_ and *C*_G2_ (to be consistent with the above estimation), as well as the tunnel resistances on source and drain side *R*_Si_, *R*_Di_ (*i* = 1,2), a condition could be found where a good match to the experimental data is achieved. Although some features are not fully reproduced, the satisfactory similarity obtained with the data shown in Figure 7b suggests that the proposed equivalent circuit can reasonably explain the observed experimental data. It can be understood, thus, that multiple QDs are easily formed in such codoped nanoscale SOI channels, giving rise to multiple current peaks due to SET transport.

**Figure 8:**
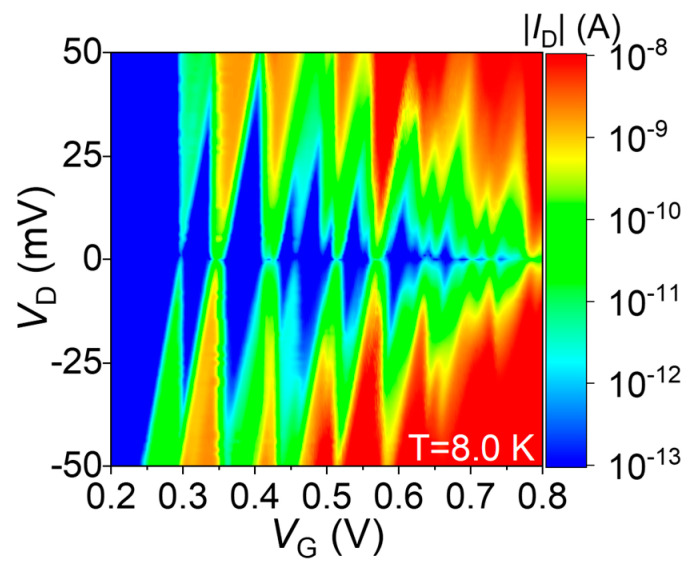
Stability diagram (plot of |*I*_D_| in the *V*_G_-*V*_D_ space) for the same codoped SOI-FET as in Figure 7, at *T* ≈ 8.0 K. Low-current diamond-shaped regions are observed (in blue) for |*I*_D_| ≤ 100 fA.

For further clarification, the stability diagram (the plot of |*I*_D_| in the space defined by the gate voltage *V*_G_ and source-drain bias *V*_D_) for this codoped SOI-FET is shown in Figure 8. Diamond-shaped low-current regions (effectively noise-level current, |*I*_D_| ≤ 100 fA) can be observed successively from *V*_G_ ≥ 0.3 V, with the first few such regions being closed, followed by overlapping diamond-shaped regions at higher *V*_G_. Although there is a trend for the height of these regions to becomes smaller as *V*_G_ is increased, alternating heights can also be observed, suggesting again the possibility of two different paths controlling the SET transport.

The model described in Figure 7 holds also for a basic interpretation of the stability diagram shown in Figure 8. A more detailed interpretation of the example results presented in this section will be provided in the Discussion section.

It should also be noted that such transistors (i.e., exhibiting SET features) can be found with relatively high yield in this batch of codoped SOI-FETs as compared to *n*-type SOI-FETs of comparable dimensions [41]. This suggests a certain impact due to the introduction of B-acceptors on the background of P-donors (for both types of devices, doped at high concentrations, *N*_D_ ≈ 2.0 × 10^20^ cm^−3^ for the codoped SOI-FETs and, respectively, *N*_D_ ≈ 1.8 × 10^20^ cm^−3^ for the SOI-FETs doped only with P-donors). However, further studies are necessary to fully clarify the interplay between the distributions of P-donors and B-acceptors for the formation of QDs in such codoped nanoscale channels.

## 4. Discussion and Outlook

In the preceding sections, the fabrication steps of codoped nanoscale SOI devices were presented in parallel for both diodes and transistors. The processes used for the two types of devices are basically the same, except for the need for doping masks when forming *pn* (and *pin*) diodes and, respectively, of the addition of a top gate for the case of SOI-FETs. This work suggests that a comparison between the two types of devices, when fabricated using such similar techniques, can allow a deeper understanding of the physics of codoping, observed by quantum tunneling either from single-band transport or band-to-band transport. Most importantly for the purpose expressed in this work, the single-charge tunneling mechanism can be evaluated (in particular at low temperature) in nanoscale depletion layers of *p*^+^*n*^+^ diodes or nanoscale channels of transistors.

The data shown in Figure 4, for a *p*^+^*n*^+^ diode, are an example of a device that works basically as an Esaki (tunnel) diode, exhibiting NDC (and excess current features) in the forward-bias region, as well as high BTBT current in the reverse-bias region. These are telltale signatures of the Esaki diodes [45,46]. It has also been reported that the phonon-assisted BTBT is preserved even in nanowire Esaki diodes [47]. In our previous work on *p*^+^*n*^+^ diodes of comparable dimensions to the ones presented here, we reported that, despite the fact that quantum confinement in the thin SOI layers starts to be observed as discrete energy states, phonons are still contributing significantly to BTBT transport [39,40]. These results suggest that Si preserves its indirect-bandgap nature in such dimensions (still larger than 10 nm), while effects of dielectric and quantum confinement on the band nature and dopant states would be expected in much smaller scales (<10 nm) [48]. Despite this typical behavior, applying *V*_sub_ as a back gate opens a (low-current) gap at low biases, as shown in Figure 5 and, more clearly, in the stability diagram plotted in Figure 6. Two main reasons can be considered for such a gap.

First, this can be due to an increase in the resistance of the top-Si film since *V*_sub_ is a macroscopic gate, affecting a wider area in the top-Si layer, not only the depletion layer. At least, it is reasonable to assume that different polarities of *V*_sub_ can induce the accumulation or the depletion of electrons or holes in different parts of the diode. The region that is most likely to be affected by *V*_sub_ is the codoped (overlap) region, which is located to the right of the *pn* junction itself, since this region must be thinner and narrower due to the double-doping process. In addition, the effective doping concentration is also lower (if assumed to be |*N*_D_ − *N*_A_|) as compared to the non-overlapped parts of the leads. Since this region is effectively *n*-type (*N*_D_ > *N*_A_ for this case), negative *V*_sub_ would induce the depletion of electrons, thus increasing the resistance. This may reasonably explain the sudden change occurring at large negative *V*_sub_, although the change is rather systematic (linear) up to that point (see Figure 5a]. For positive *V*_sub_, electrons are accumulated in this region, making it less resistive and, thus, allowing higher currents to flow. Although this is generally consistent with the observation in Figure 6, this model cannot explain the diamond-shaped regions clearly noticeable in the positive-*V*_sub_ regime (see Figure 5b and Figure 6).

Therefore, a second and more likely explanation for the peculiar evolution of the low-bias gap is the phenomenon of Coulomb blockade, which assumes that one (or just a few) QDs significantly control BTBT transport at such low biases [40]. As assumed in our previous works, it is more likely that clusters of P-donors (“molecule”-like structures or even nanoscale “domains”) are formed near the *n*-type edge of the depletion layer. This is due to the fact that *N*_D_ is higher than *N*_A_, thus favoring such *n*-type cluster formation. Assuming this model, the *V*_sub_ effect can be explained as follows. At negative *V*_sub_, the donor-cluster working as a QD is empty (no electrons are trapped into it), which is consistent with the general situation in a depletion layer (no captured carriers, in principle). As *V*_sub_ is increased in the negative direction, the gap broadens rapidly because larger and larger *V*_p_ would be needed to realign the QD’s ground state into the BTBT transport window to allow the current to start again. At positive *V*_sub_, however, electrons can be added one by one into the QD(s) and BTBT transport becomes dominated by Coulomb blockade. The observation of only two Coulomb diamonds (and even these with some sub-structure) is reasonable considering the large electric field present across the depletion layer; such a condition may only allow the addition of two electrons before the tunnel barrier becomes negligible. The critical tunnel barrier is likely on the side towards the *n*-lead.

In reality, there may be contributions from both models described above. However, Coulomb blockade and single-charge tunneling remain the only phenomena that can reasonably explain the diamond-shaped regions. To fully clarify the impact of the design parameters, in particular the role of codoping, in defining the QDs that allow SET transport, further study is necessary by gradually changing the device dimensions, doping concentrations, lengths of the overlapped region, and temperature. This remains as a future study.

The codoped SOI-FETs (practically designed as codoped junctionless transistors) are a more straightforward case of study because transport does not involve both conduction and valence bands. As such, the indirect-bandgap nature of Si does not have to be specifically considered in the interpretation of transport. However, it should be noted that the distributions of P-donors and B-acceptors are most likely not correlated to each other, meaning that there could be preferential positions for opposite-polarity dopants (near interfaces or, respectively, within the nanowires), which would allow the formation of *n*-type paths and *p*-type paths within the same channel. To elucidate such a possibility, further study is needed, involving tuning of the vertical electric field (e.g., using both *V*_sub_ and *V*_G_ in an appropriate configuration) [49]. This study also remains to be reported later.

Here, we focus on the electrical characteristics presented in Figure 7, in comparison with a Coulomb-blockade simulation result, and on the stability diagram shown in Figure 8. These results illustrate that nanoscale codoped SOI-FETs can contain in their channels one (or just a few) QD(s) that provide a platform for the observation of SET transport. There are also several possibilities to explain the occurrence of the QDs in such channels.

First, one can expect that high doping with both P-donors and B-acceptors, combined with the patterning-induced fine roughness (surface roughness and/or line edge roughness) of the nanoscale-channel can create potential undulations within the channel. Such potential undulations have been reported to work as QDs when the surface was intentionally roughened by chemical processing [33,34] or unintentional lack of control. Some degree of roughness was observed indeed in several devices from this batch, but mostly in the devices with longer channels, in which unavoidable process fluctuations can introduce some modulation of the nanowire thickness and/or width. However, the devices presented here have very short channels (nominally designed as point contacts), which drastically minimizes the impact of such roughness. Furthermore, the patterning and doping processes were carried out with specific care to protect the top surface and side walls of the nanostructures, as described earlier.

Therefore, a more reasonable explanation for the formation of the QDs is the random distribution of dopants within the Si nanoscale channel. In our previous work [41], we illustrated statistically that even highly-doped channels may contain isolated QDs (i.e., with the “metallic” conduction paths cut off) due to the combined effect of the randomness of the dopant distribution and nanoscale dimensionality of the channel. In that case, P-donors were the only dopants introduced in the channel, at a concentration comparable to the one used in the codoped SOI-FETs. Considering that, in these codoped SOI-FETs, a significant fraction of the P-donors is effectively compensated by the B-acceptors (here, introduced in a ratio of roughly 1:3~1:4), the probability of finding isolated QDs in the codoped channels is enhanced. This can explain the larger yield of SET devices observed in the codoped-transistor batch.

Returning to the analysis of the periods between consecutive current peaks (ascribed to two different QDs, as described earlier in Figure 7), one can evaluate the gate capacitances *C*_G_ and then the area of the QDs as seen from the gate. Assuming a simple parallel-plate model, with *C*_G_ = ε_0_ε_ox_*A*/*t*_ox_ (ε_0_—vacuum permittivity; ε_ox_ = 3.9—relative permittivity of SiO_2_; *A*—area of the QD; *t*_ox_ = 10 nm—gate oxide thickness), the areas of the two QDs are estimated to be approximately 700 nm^2^ (for the smaller QD) and 1300 nm^2^ (for the larger QD). The values are acceptable to fit into the nanoscale channel, estimated from the SEM image (as shown in Figure 3c) to have dimensions of roughly *L* ≈ 100 ± 20 nm and *W* ≈ 30 ± 10 nm.

Full elucidation of the role of the counter-dopants (here, B-acceptors) in the formation of the QDs cannot be expected only from electrical characterization. Techniques that can map the surface potential of the codoped nanoscale channels, such as Kelvin probe force microscopy (KPFM) [50,51], are promising for revealing the domain formation in such channels, providing further insights into the interplay between donors and acceptors under different biasing. Further exploration of this interplay in codoped transistors can be done by changing systematically the channel dimensions (not only width and length, but also thickness), doping concentrations, processing conditions, and, eventually, by introducing selective nanoscale doping to enhance the controllability of the QD formation. This work aims to trigger more interest in these directions of research in the future.

The experimental results presented in this work provide examples of what phenomena can be revealed in codoped devices, illustrating the potential of codoped thin (and ultrathin) layers of Si (here, SOI) as a platform for observation and analysis of single-charge tunneling functionalities. It is worth noting that the study can be scaled down to the fundamental analysis of the interplay between one P-donor and one B-acceptor (or just a few atoms of each type) by first-principles simulations. Such analyses have been carried out extensively for Si nanocrystals [52,53,54] mainly with the aim of demonstrating their applicability to enhancing photoluminescence (PL). Only recently, studies have started to explore the effects of different configurations of counter-dopants in nanowire transistors [55]. It is expected that research at such fundamental level can reveal exciting new physics arising from the atomic-level counterparts of the nanoscopic devices presented here, diodes and transistors.

## 5. Conclusions

In summary, this work provided a brief overview of the fabrication and low-temperature operation of codoped silicon nanodevices (doped with both P-donors and B-acceptors), with a few experimental results demonstrating the potential for single-electron (single-charge) tunneling functionalities. The low-dimensional devices can be formed using CMOS-compatible techniques in silicon-on-insulator (SOI) films, with comparable processes for the formation of SOI diodes and SOI transistors. Codoping plays a key role either in the depletion layer (in the case of diodes) or in the nanoscale (point-contact-design) channel (in the case of transistors).

SOI *pn* diodes with high doping can behave, basically, as Esaki diodes at low temperature, but they are also sensitive to the substrate voltage. The dependence on *V*_sub_ reveals in some devices the opening of Coulomb diamonds, which suggests that single-charge tunneling may occur in such devices. Codoped SOI transistors are designed practically as junctionless transistors, but their low-temperature characteristics clearly exhibit single-electron tunneling features (current peaks and Coulomb diamonds in the stability diagram). The interpretation is straightforward based on the orthodox theory of Coulomb blockade, suggesting that such codoped channels are promising platforms to observe and analyze single-electron tunneling functionalities based on dopants.

Further developments in the ability to engineer the electronic properties of codoped silicon at the nanoscale will open new opportunities for studying in more depth quantum phenomena, such as single-electron tunneling. It is important to note that, while this article provides a glimpse into the capabilities of codoped silicon nanodevices, further systematic studies are required to fully understand and harness their potential. Future research efforts should focus on exploring the underlying mechanisms of single-electron tunneling in codoped silicon, as well as on optimizing device fabrication techniques for improving device yield.

In conclusion, the experimental results presented here highlight the exciting possibilities that codoped silicon nanodevices hold for fundamental physics exploration. Continued research in this field will contribute to the advancement of nanoelectronics and pave the way for donor-acceptor-based functionalities and novel device architectures.

## Data Availability

Data is available upon reasonable request.
